# Developing an Open Multi-Domain Assessment Battery Using Laboratory Tasks for Children in Low- and Middle-Income Countries

**DOI:** 10.1162/OPMI.a.369

**Published:** 2026-09-05

**Authors:** Joseph R. Coffey, Elizabeth Spelke

**Affiliations:** Laboratoire de Sciences Cognitives et de Psycholinguistique, Département d’études cognitives, ENS, EHESS, CNRS, PSL University; Laboratory for Developmental Studies, Department of Psychology, Harvard University

**Keywords:** validation, intervention, LMIC, development, Ghana, cognitive science, field experiment

## Abstract

Recent years have seen growing interest in the use of randomized designs to evaluate the impacts of interventions on early child development in low- and middle-income countries. Measuring these impacts requires assessments that can track development prior to school entry in settings that are often noisy and outdoors. Drawing from research in developmental cognitive science, we created an open multi-domain assessment battery for testing children in low- and middle-income countries consisting of tasks based on experimental paradigms used in laboratory settings. These tasks were designed to target abilities that emerge across cultures early in life and predict later performance on related skills in adulthood. This battery was administered to 1435 Ghanaian children whose parent previously took part in a randomized controlled field experiment on the impacts of secondary education on their children's development, measuring performance on tasks at ages 1.5, 3.5, 5.5, and 7.5 years. Starting at age 3.5, children’s responses to the tasks were internally consistent and above chance, with overall test results predictive of later performance. There was strong evidence of validity both for tasks in numeracy and spatial reasoning, which were designed to tap into culturally invariant cognitive capacities, as well as those in language and executive functioning, which required more context-specific adaptation. In contrast, there was little evidence of validity in infant tasks or tasks focused on intuitive physics or mental state reasoning. Nevertheless, our results suggest that our approach to assessment design is effective at producing tasks that are internally consistent and sensitive to individual differences within cohort. By making our testing materials and data freely accessible, we hope to provide a foundation for the development of tools for studying cognitive development in field settings.

## INTRODUCTION

Early childhood is a critical period for healthy cognitive development and has attracted broad interest internationally, particularly in low- and middle-income countries (LMICs) where poverty and other risk factors can disrupt development and later achievement in school (ASER, 2023; Bendini & Devercelli, 2022; Black et al., 2017; Sania et al., 2019). To address these problems, development economists have partnered with cognitive scientists to develop and evaluate interventions to enhance children’s cognitive development and school preparedness globally (e.g., Dean & Jayachandran, 2020; Dillon et al., 2017; Dupas et al., 2025). To assess their efficacy, these interventions require measures of child development that can be administered in a multitude of cultural contexts and capture individual differences from infancy to school age. Without such assessments, it is difficult to determine whether an intervention can generalize from one setting and population to others.

Researchers have found some success adapting assessments that were created and normed in the U.S. (for a useful overview of assessment adaptation and development, see Fernald et al., 2017). For example, the Ages and Stages Questionnaire has been adapted for use across many different countries (Squires & Bricker, 2009; e.g., in Muthusamy et al., 2022). There have been efforts in recent years to design novel assessments that directly address the needs of researchers working in LMICs. Some assessments come in the form of structured questionnaires given to a child’s primary caregiver. For example, the Caregiver Reported Early Development Instruments (CREDI) is a cross-nationally normed caregiver survey that tracks language, cognition, motor skills, social reasoning, and mental health of children up to the age of three (McCoy et al., 2018). Like the ASQ, the CREDI has also been found to predict individual differences in child development across a range of cultural settings (e.g., McCoy et al., 2021; Waldman et al., 2021). Other caregiver reports, such as the Kilifi Developmental Inventory (Abubakar et al., 2008), were designed not as cross-nationally valid caregiver reports, but as diagnostic measures adapted directly for use in particular cultures.

Direct assessments of children are more difficult to administer than caregiver reports, but they have desirable features: whereas caregiver reports use adult accounts of observed behavior to track children’s skills and knowledge, direct assessments task children to use those skills and knowledge in novel contexts. In addition, researchers also often aim to relate children’s developmental outcomes to parent-reported survey information, such as the frequency of cognitively stimulating activities in the home. For this purpose, however, using caregiver-reported assessments as the measure of children’s abilities risks producing circular conclusions, because caregivers who engage in more activities with their children may be more attuned to their children’s abilities. Finally, for educational interventions that target parents’ knowledge or attitudes regarding child development, caregiver reports might not reflect genuine changes in children’s development, but instead parents’ newfound attentiveness towards their children’s attained milestones. For these reasons, direct cognitive assessments of children may be particularly useful in measuring cognitive development in LMICs, where many children spend more time with their siblings and peers than with their primary caregivers, and thus where developmental benchmarks may not be actively tracked by adults. As a result, direct assessment has proven appealing for many researchers working in these contexts.

There have been a number of different approaches to direct child assessment in LMICs. Often researchers will adapt existing assessments to local contexts. For instance, in a meta-analysis of 21 studies relating early life risk factors to subsequent cognitive development in LMICs, Sania et al. (2019) found that 13 of these studies used adaptations of the Bayley Scales of Infant and Toddler Development (Bayley, 2005). More recently, new assessments specifically designed for use in LMICs have been developed. The International Development and Early Learning Assessment (or IDELA) is a direct assessment targeted at children between the ages of 3.5 and 6.5 years (Pisani et al., 2018); it has been adapted for use in 76 countries and translated into more than 50 languages. The East Asia-Pacific Early Child Development Scales (EAP-ECDS) has also been validated for use for children from 3 to 6 years of age in four East Asian countries and addresses regionally specific skills, such as cultural knowledge (Rao et al., 2019).

There are a number of unique challenges involved in creating assessments of cognitive development for use in LMICs. Children’s cognitive development has been studied most intensively in urbanized, high-income Western countries. Since at least the 1930’s, developmental scientists have noted that many methods for testing mental abilities in those countries are inappropriate for use in other cultural settings. For example, children who have less exposure to formal schooling use different strategies for generalization or logical reasoning that may produce an incorrect response on a standard assessment (e.g., Greenfield & Bruner, 1966 in Senegal; Scribner & Cole, 1973 in Liberia; Luria, 1976 in Uzbekistan). As a result, the developmental trajectory of certain cognitive abilities can differ greatly across cultures. Kroupin et al. (2025) find no evidence of universal developmental trends in executive function tasks across schooled and non-schooled populations, with each group exhibiting distinct pattern of development. Because children’s approach to learning is shaped by the values and practices of their cultures (e.g., Legare, 2019; Rogoff et al., 1993), different approaches to socialization may also produce vastly different patterns of development. For instance, Lamm et al. (2018) found that 4-year-old Nso children from Cameroon performed at ceiling in a delayed gratification task while their German peers were highly varied in their success. This difference was attributed to socialization practices that encourage Nso children to be socially conscious and deferential, and German children to be psychologically autonomous and independent. As a result of such differences, tasks that are used to measure differences in the pace of children’s development or predict their academic success could be completely ineffective in certain settings. Large-scale field studies in LMICs must also contend with subject pools that include children from multiple distinct communities, each with different cultural attitudes and experience with schooling.

One approach to these challenges is to leverage lab-based experimental paradigms that have been used to study cognitive abilities present in infancy and attested by research on adults with minimal education living in remote communities in diverse cultures (e.g., Dehaene et al., 2006; Jara-Ettinger et al., 2017; Pica et al., 2004). These paradigms have been pivotal in establishing the existence of cognitive capacities that emerge and function in the same way across a diverse range of early life contexts, and that relate to the development of school-related skills later in life. For instance, experimental research has found that before children learn arithmetic, they possess systems for representing exact, small numbers of objects (Feigenson et al., 2002) and the relative sizes of larger sets of objects with approximate precision (Izard et al., 2008), as do adults in remote cultures with little to no formal education (Pica et al., 2004). Performance on related tasks in infancy has been found to relate to their later performance in school mathematics (e.g., Feigenson et al., 2013; Gilmore et al., 2007; Halberda et al., 2008; Starr et al., 2013). There is compelling evidence that this capacity is universal, as it has been found not only in urbanized Western contexts, but also in preliterate hunter-gatherer societies whose languages lack formal number systems (Jara-Ettinger et al., 2017; Pica et al., 2004). Likewise, children represent object kinds based on their skeletal shapes and the lengths and angles at which their parts conjoin (Dehaene et al., 2006), and they can navigate through known environments both with and without maps (Shusterman et al., 2008), well before they study geometry in school. These capacities predict later performance on school-related geometric tasks, as well as intuitive judgements of geometric properties (Dillon & Spelke, 2018; Giofré et al., 2013), and are present in children from preliterate societies with no exposure to formal geometry or map-based navigation (Dehaene et al., 2006).

These paradigms and the abilities they target are highly relevant for development economists and other researchers working in LMICs who wish to track individual differences in the pace of cognitive development following intervention, especially those that pertain to academic preparedness. They also promise to vastly increase the scale and generalizability of research in cognitive science. Despite making up only 15% of the world’s population, Nielsen et al. (2017) found that over 92% of studies in developmental cognitive science were conducted either in the U.S., in other English-speaking countries, or in Europe. By adapting these methods for use in randomized field experiments in LMICs, in which many of the most revealing tasks shedding light on children’s cognitive development have not previously been used outside of the U.S. and Europe, one may increase the size and diversity of potential subject pools from dozens to thousands.

Here, we present an open multi-domain assessment battery designed to measure children’s development in LMICs. The battery consists of 27 tasks that are meant to target one of five broad domains of cognition: Language, Numeracy, Spatial Reasoning, Mental-State Reasoning, and Executive Function. The battery is designed to be administered to children ranging from 14- to 99-months-old, who receive a subset of these tasks adjusted for their age. The tasks range from tests of implicit cognition based directly on experimental paradigms used in cognitive development research (e.g., approximate number comparison, naïve physics), to tests of school-related skills previously employed in other cross-national assessments (e.g., reading comprehension, identifying shapes). The aim of the battery is not to establish international norms for typical cognitive development, which carries the risk stigmatizing communities where they may not be contextually appropriate (Scheidecker et al., 2021), but rather to provide researchers working in diverse settings with tools for measuring individual differences within a population. The battery materials and scripts, as well as performance data for children who have been administered the battery, are available through the Inter-University Consortium for Political and Social Research (ICPSR). We hope that the accessibility of this data will motivate future task development and the adaptation of the battery for new settings.

The battery was originally designed for use with children growing up in Ghana. Ghana is located in West Africa, which has the fastest growing child population in the world, with the number of children aged 0–4 projected to increase from 65 million to nearly 99 million by 2050 (Lam et al., 2019). Among other countries in the region, Ghana has the highest rates of enrollment in early childhood education programs, with 80% of eligible children enrolled in preschool as of 2016 (Ghana Ministry of Education, 2016). Early childhood education remains a significant priority in Ghana, where in spite of high enrollment rates, many children still fail to meet key developmental milestones (McCoy et al., 2016).

In this paper, we examine data from Ghanaian children whose caregivers participated in a randomized field experiment in 2008, which measured the effects of senior high school scholarships on a range of educational and life outcomes (Duflo et al., 2021). This experiment found that the intervention increased parents’ years of education and their likelihood of enrolling in post-secondary education, relative to peers who received no scholarships. Due to a national shortage of white collar jobs at the time of their graduation, the income levels of the treated and untreated parents did not differ. Starting in 2016, surveyors began assessing the children of these participants to investigate potential downstream effects of parent education on health and cognitive development using the previously described assessment battery (Duflo et al., 2024). Because children’s performance on related tasks has been previously associated with household education (e.g., numeracy/spatial reasoning: Bachman et al., 2022; mental-state reasoning: Ebert et al., 2017), they believed the battery would be able to capture the potential effects of the intervention on children’s cognitive abilities. They found that children of mothers in the treatment condition, who were offered scholarships that covered the cost of high school, scored more highly on the assessment in the two older age cohorts (5.5-year-olds and 7.5-year-olds) but not in the two younger age cohorts (1.5-year-olds and 3.5-year-olds). These associations in turn were correlated with female caregivers’ engagement in cognitively stimulating behaviors with children, such as reading and storytelling, as well as their increased practicing of preventative health behaviors (e.g., vaccinations and regular medical check-ups).

While these findings suggest that the battery is sensitive to caregiver education, possibly via its effects on children’s early learning environment, no formal measures of construct validity or measurement reliability have been reported. A recent review of the American Economic Association’s registry of randomized controlled trials found that of 237 registered studies focused on measuring children’s school-related skills from ages 3 to 18, over two-thirds provided no evidence of validity or reliability for their outcome measures (Macours et al., 2023). From our current data, it is not possible to unambiguously determine whether our tasks tap into culturally invariant cognitive development, or more contextually specific skills that are also sensitive to the parent treatment described previously. Thus, the present study aims to assess the reliability and validity of these tasks within the current testing context, to determine whether the results of the treatment reflect a genuine and meaningful effect on children’s task performance. To this end, we focus on three dimensions of assessment efficacy. First, does the battery produce responses from children that are internally consistent and above chance (measurement reliability)? Second, does performance on each task relate to performance on other tasks within the same cognitive domain (convergent validity)? And third, does performance on a task at younger ages predict performance on related tasks at older ages (predictive validity)?

## METHODS

### Participants

The present evaluation is based on data collected as part of a larger randomized controlled trial of 2,064 high school students (*n* = 1036 female) that began in 2008 (Duflo et al., 2021). These students (who are now the parents of our child participants) were drawn from households in Ashanti and Brong-Ahafo, and in the Central, Eastern, and Western regions. In Ghana, high school admission is contingent on performance on an examination taken at the end of junior high school. At the time this study began, high school attendance was not compulsory and required yearly tuition as well. All students selected for the study had qualified for high school admission based on their performance on the entrance exam but failed to enroll in high school by the deadline; over 90% cited financial constraints as the reason for not enrolling. For one third of the male and female students, full scholarships covering their tuition and course materials were paid directly to their school; and the remaining students were followed over time as controls. Since then, these participants have been surveyed yearly on various outcome measures, including their level and type of occupation, income, housing, and family arrangements, including marriage and children.

To ensure that the validity of the measures was not affected by children’s experience with formal education, which was greater for treated households (Duflo et al., 2024), we excluded children whose parents participated in the treatment condition (*n* = 712). Thus, only children whose parents participated in the no-treatment control condition of the project contributed data to the present analyses. In this group, primary caregivers reported an average of 9.28 years of education. Most caregivers reported speaking primarily some dialect of Akan to children (1,062 or 74%). Other reported languages include Nzema, Ewe, Hausa, and Ga-Dangme. Many caregivers reported that their children were exposed to multiple languages (647 or 45%), mostly commonly English (484 or 75%). 527 or 37% of households were located close to urban centers.

We measured 1435 children’s cognitive abilities within at least one of four age ranges over the course of six years (2016–2023): 14 to 25 months (mean = 18 months; *N* = 671; 322 male, 341 female, remaining unavailable); 39 to 55 months (mean = 44 months; *N* = 712; 362 male, 339 female); 60 to 83 months (mean = 66 months; *N* = 732; 357 male, 337 female), and 84 to 99 months (mean = 89 months; *N* = 471; 199 male, 194 female).^1^ These ages (hereafter, referred to as 1.5, 3.5, 5.5, and 7.5 years, respectively) were originally selected to determine if and when parents’ education had an impact on their children’s cognitive development. For example, if parents’ education had affected caregiving practices from birth, it would have been reflected in the performance of 1.5 year-olds, with possible cumulative effects throughout early development reflected in performance at 3.5 years, especially on skills that children learn early on at home, such as vocabulary. But if parental education had not changed early caregiving practices and instead impacted either parents’ willingness or ability to engage with children’s schoolwork, or children’s intrinsic motivation to succeed in school, these effects would only have become visible at 5.5 years or at 7.5 years, especially on skills that children learn in school, such as symbolic mathematics.

Children were assessed at their residence in sixteen rounds of successive testing, each lasting about 2–3 months. Each child received at most a single assessment in every age group they were eligible for during this period, contributing between one and four scores total. In total, 518 children were tested once, 479 were tested twice, 368 were tested three times, and 70 were tested four times. Because the population of families was dispersed across widely differing and often remote areas, the age range of tasks was wide, including children at different points in development. For example, the youngest age range included both 14-month-old infants who were just beginning to walk and talk, as well as 25-month-old children nearly twice their age. As a result, we would expect variability in children’s performance not only across ages but also within them. To ensure that cognitive performance was assessed relative to the child’s maturational status and experience, we followed the procedures used in the field experiment (Duflo et al., 2024) and scored cognitive performance within each age group using each participant’s age as a regressor. Thus, a 14-month-old infant and a 25-month-old infant would both be given the same tests for the 1.5-year age group, but if the number of items that they performed correctly were the same on a given test, the infants’ adjusted scores gave the younger child a higher score than the older child.

### Adapting Tasks and Materials

Descriptions of tasks administered can be found in Table 1. The selection of the five core domains overlaps with the structure of many other assessments and questionnaires that have since been used in Ghana, including the CREDI (McCoy et al., 2018) and IDELA (Pisani et al., 2022; Wolf & McCoy, 2019), focusing on abilities that emerge early in development and remain present throughout adolescence. In addition, a subset of these tasks in numeracy, spatial reasoning, and mental-state reasoning were previously used in a classroom intervention in India, focused on improving children’s math learning by training implicit numerical and geometrical intuitions (Dillon et al., 2017). We used these materials as a basis for adaptation in Ghana.

**Table 1.** Descriptions of each task administered organized by domain and the number of trials at each age.

**Domain**	**Description**	**1.5Y**	**3.5Y**	**5.5Y**	**7.5Y**
Language					
CDI	Caregiver reported vocabulary checklist	88	–	–	–
Vocabulary	Match spoken words to pictures	–	32	32	32
Reading	Recognize print letters, words, and sentences	–	–	–	12
Numeracy					
Number Compare	Identify the larger of two piles of toys	3	–	–	–
Verbal Number	Compare exact quantities and basic arithmetic	–	7	12	6
Panamath	Compare approximate value of large dot arrays	–	12	9	18
Point to Number	Match verbal numbers to:	–	–	–	–
(Symbols)	(printed numerals)	–	4	6	6
(Objects)	(small arrays of objects equal in magnitude)	–	5	9	–
Spatial Reasoning					
Block Reproduction	Build small block towers with the surveyor	–	7	–	–
Geometric Intruder	Find the shape that is different than others	–	6	7	6
Map Reading	Place object on a location on a village map	–	7	11	–
Verbal Geometry	Identify parts of different shapes	–	–	6	9
Naïve Physics	Predicting the weight of falling objects	–	–	3	–
Point to Shape	Identify shapes by name	–	–	6	11
Mental State Reasoning					
Point to Emotion	Match emotion word to correct face in array	–	3	6	–
Gaze Direction	Find which object a face is looking at	–	3	–	–
Emotion Intruder	Find the face with a different emotion	–	–	–	6
Gaze Intruder	Find the face with a different gaze direction	–	–	–	8
Theory of Mind	False-belief or ‘Sally Anne’ task	–	–	4	13
Executive Function					
Working Memory	Recall the location of a hidden object	3	–	–	–
Attention Switching	Refocus on a hidden object after a misdirection	2	4	–	–
Mental Simulation	Track the movement of a hidden object	2	4	–	–
Baseline EF	Press button on same side as stimuli	–	–	10	10
Response Inhibition	Press button on opposite side of stimuli	–	–	10	10
Task Switching	Switching between two button press rules	–	–	10	10
Impulsivity	Quickly select matching object near similar ones	–	–	–	7

A significant barrier to the development of assessments in LMICs is establishing their ecological validity, or their relationship to abilities children employ in everyday life, in a field setting, which we define for our purposes as a non-laboratory outdoor setting close to the child’s residence. Threats to ecological validity can come in many forms. Children might pass or fail a test for reasons unrelated to their abilities if they lose interest or motivation during testing or misinterpret the surveyor’s instructions. They might also fail tasks that contain references to materials outside of their cultural context, or that ask them to engage in games or routines they are unfamiliar or uncomfortable with (see Jukes et al., 2024 for further discussion). Given these challenges, the field experiment was preceded by a year-long period of pilot testing in 2016 to ensure tasks were adapted to the Ghanaian context. Further piloting of changes to the measures was conducted as problems arose thereafter. We focused on five basic conditions, drawn from the many factors that can help to achieve ecological validity.

First, we recruited local surveyors from in and around Ghana’s capital, Accra, to help us develop and administer the assessments. Throughout the study, 31 surveyors were involved in testing. Most surveyors were involved in more than one phase of testing (25) and tested children in more than one age group (29). None had prior educational or practical experience in psychology or early childhood development and thus were unlikely to have the same preconceptions about task design or children’s cognitive abilities as would trained researchers. The initial test battery was designed to be implemented with minimal training, which took place the week before deployment into the field, with retraining sessions occurring between rounds of testing. During these periods, surveyors also reported any problems they encountered in the field so that testing materials could be adjusted. Surveyors were blind to whether families belonged to the treatment or control conditions to prevent bias.

Second, we aimed to ensure children felt comfortable with how testing was conducted. Many children living in remote areas of Ghana rarely see adults from outside their community and so may be frightened by or unresponsive to unfamiliar surveyors who approach them, offer them games, and ask them direct questions. Thus, we relied on laypeople drawn from local communities to put children at ease and render them more receptive to the tasks the surveyor presented to them. We also encouraged surveyors to elicit assistance from others in the community: for example, by having the child’s caregiver or older sibling play the game to be used in the assessment before it was played by the child.

Third, we worked to make children feel able and motivated to take part in the tasks. In addition to making the assessment materials engaging, this also meant ensuring that children were able to understand task instructions and give appropriate answers. As a result, tasks were designed to require minimal verbal responses, relying mostly on gaze or gesture. Tasks were introduced with 1 to 2 practice trials, which can reveal children’s misinterpretations of task demands and allow surveyors to deliver feedback to correct their understanding. Local surveyors also noted patterns of chance responding, location biases in children’s pointing, or systematic misunderstandings of tasks during piloting, allowing us to adjust problematic tasks.

Fourth, we ensured that task materials contained images and routines familiar to the children. Although the tasks target abilities that have been shown to emerge across cultures, in practice assessments often contain references to things specific to a cultural context (places, animals, objects) or are based on games or routines that are familiar to some children but not others. For this reason, we worked with our local surveyors to ensure that the battery contained culturally relevant materials and tasks that were based on routines familiar to the children being tested (e.g., replacing English food words like *candy* or *cookie* with local equivalents like *toffee* and *biscuit*, ensuring animals used in stimuli were recognizable to children, and using the layout of the child’s village for map navigation tasks). Because there are additional challenges involved with administering vocabulary tests in multiple languages, an in-depth validation of the language measures was conducted separately, which found that caregiver responses had high internal consistency and most of the items were effective at distinguishing children based on suggested language ability. The validation also failed to find any systematic differences in performance based on the language the checklist was administered in, suggesting no bias based on parent English proficiency (Coffey et al., 2025).

Finally, we created task materials that were practical in the environments in which testing took place. Children were often living in communities located hours away from urban areas. Within these communities, surveys usually needed to take place outside due to limited indoor space. Thus, assessment materials were designed to be portable, durable, and usable both indoors and outdoors. For the 5.5- and 7.5-year-old children, tests took place on the laptops which the surveyors also used to log parental survey data. However, during piloting, surveyors reported a large number of 1.5- and 3.5-year-old children who were unable to complete screen-based assessments. As a result, small, lightweight plastic objects (e.g., Legos, miniature animals, plastic cups) or laminated pictures were used in lieu of laptops.

### Procedure

At 1.5 and 3.5 years, tasks were administered by a live surveyor who faced the child, presented objects (cups, small toys) on a table between them, explained each task, and provided feedback on practice trials. Children sat with one or more family members (usually the primary caregiver) who were asked not to interfere with or help them. They responded either verbally or by pointing to a test object or image. At 5.5 and 7.5 years, children sat in a chair at a distance from all family members, and tasks were presented on a computer by a surveyor who drew children’s attention to the screen, provided explanations and feedback on practice trials, and posed the test questions. Children responded either verbally or by keyboard press. At all ages, children received feedback on practice trials but no informative feedback on test trials.

### Analysis

All analyses were conducted in R (v 4.4.1) (R Core Team, 2024). Given the wide range of ages within each group, we followed the procedures used in the field experiment and scored task performance using each participant’s age as a regressor to control for within-age-group variability. To assess the sensitivity and validity of these tests, we analyzed children’s average overall score, their average score within each domain, and their score on each task. Children who did not respond to any trials of a given task were excluded from analyses involving that task (the final *N* for each task is given in Supplementary Materials). Within tasks, non-responses to trials were counted as incorrect answers.

For measurement validity, we measured the internal consistency of task performance and the rates of random or incorrect responses. For internal consistency, we calculated Cronbach’s alpha, setting an acceptability cutoff at 0.6 (Taber, 2018). For our measure of random responses, we examined the distribution of raw scores across the tasks. We counted the number of children performing above chance, calculated by comparing each child’s score to the average chance performance on each task. We postulated that for each task to have sufficient variance to find individual differences between children, there should be at least twice as many children above chance as below chance.

Due to the length of the battery and number of participants our surveyors needed to reach within a single round of testing, we were not able to present children with other assessments (e.g., IDELA) for comparison. To assess convergent validity in lieu of this, we measured the correlations in performance between every pair of tasks within a domain. For example, children who score highly on tests of sensitivity to approximate numerical magnitudes also tend to score higher on tests of knowledge of numbers and arithmetic (Feigenson et al., 2013). We tracked whether task performance was correlated with performance on at least half of the other tasks tapping knowledge in the same domain.

For predictive validity, we examined correlations between children’s earlier scores to their scores on the same or similar tasks assessed at later ages. For example, children who score highly on tests of sensitivity to number in infancy also tend to score higher on such tests at later ages (Starr et al., 2013). Thus, if the present battery accurately assessed a child’s sensitivity to number, then performance at each age should predict their performance at the next age group. As in our convergent validity analysis, we tracked whether task performance at earlier ages predicted later performance on at least half of the related tasks at later ages. Predictive validity was only calculated for children who had been assessed twice in two different age groups. For this purpose, data were available from 344 children with measures at both 1.5 and 3.5 years, 413 children at 3.5 and 5.5 years, and 360 children at 5.5 and 7.5 years.

## RESULTS

Summaries of each analysis are given in Tables 2–5 (for detailed tables, see Supplementary Materials). Above Chance indicates the percentage of children who perform above chance (* at least 2/3 of participants performing above chance). Internal Consistency indicates Cronbach’s alpha (* *α* above 0.6). Convergent Validity indicates the mean correlation with other tasks assessing the same cognitive ability, as well the as range when applicable (* significantly and positively correlated with at least half of other tasks). Predictive Validity indicates the correlation with tasks in the next age group that target the same cognitive ability (means and range given at task-level) * significantly and positively correlated with at least half of the related tasks given to the next age group). Significance thresholds (*a*), which were used to determine the statistical relevance of correlations, were corrected for number of comparisons (*m*) using Bonferroni correction (*a*/*m*). Dashes indicate where analyses could not be conducted for these tasks, because the ability they targeted was not assessed at successive ages. For example, no analysis of Predictive Validity could be conducted for 7.5-year-olds.

**Table 2.** Evaluation of the 1.5-year-old assessment battery organized by domain (bolded) and task. Asterisks indicate tasks that meet thresholds for measurement reliability (above chance performance), internal consistency (Cronbach’s alpha), convergent validity (*p* < 0.013), and predictive validity (*p* < 0.03 for Domain and Overall; *p* < 0.006 for Task).

**Domain**	**Above Chance (%)**	**Consistency (*α*)**	**Convergent (r)**	**Predictive (r)**
**Language**	–	0.99*	–	0.2*
Understands	–	–	0.7*	0.21*
Says	–	–	0.7*	0.14*
**Numeracy**	64%	−0.37	–	–
Number Count	64%	−0.37	–	–
**Executive Function**	78%*	0.13	–	−0.13
Attention Switching	37%	−0.11	0.13*	−0.07*
Mental Simulation	40%	−0.32	0.11*	−0.15*
Working Memory	79%*	−0.13	0.1	0
**Overall**	46%	0.16	–	−0.07

**Table 3.** Evaluation of the 3.5-year-old assessment battery organized by domain (bolded) and task. Asterisks indicate tasks that meet thresholds for measurement reliability (above chance performance), internal consistency (Cronbach’s alpha), convergent validity (*p* < 0.005), and predictive validity (*p* < 0.01 for Domain and Overall; *p* < 0.001 for Task).

**Domain**	**Above Chance(%)**	**Consistency (*α*)**	**Convergent (r)**	**Predictive (r)**
**Language**	99%*	0.81*	–	0.26*
Vocabulary	99%*	0.81*	–	0.26*
**Numeracy**	81%*	0.66*	–	0.22*
Panamath	59%	0.55	0.18*	0.1
Verbal Number Question	87%*	0.28	0.19*	0.02
Point Number (Symbol)	83%*	0.29	0.19*	0.07
Point Number (Object)	40%	0.56	0.24*	0.18*
**Spatial Reasoning**	94%*	0.36	–	0.21*
Geometric Intruder	68%*	0.22	0.12*	0.08
Map Reading	89%*	0.3	0.12*	0.06
Block Reproduction	–	–	0.16*	0.12
**Mental State Reasoning**	65%	0	–	0.1
Point Emotion	34%	−0.02	0.09	0.06
Gaze Direction	60%	−0.31	0.09	0.03
**Executive Function**	97%*	0.43	–	0.17*
Attention Switching	96%*	0.39	0.12	0.07
Mental Simulation	82%*	0.37	0.12	0.08
**Overall**	99%*	0.84*	–	0.31*

**Table 4.** Evaluation of the 5.5-year-old assessment battery organized by domain (bolded) and task. Asterisks indicate tasks that meet thresholds for measurement reliability (above chance performance), internal consistency (Cronbach’s alpha), convergent validity (*p* < 0.003), and predictive validity (*p* < 0.01 for Domain and Overall; *p* < 0.001 for Task).

**Domain**	**Above Chance (%)**	**Consistency (*α*)**	**Convergent (r)**	**Predictive (r)**
**Language**	100%*	0.79*	–	0.42*
Vocabulary	100%*	0.79*	–	0.36*
**Numeracy**	88%*	0.8*	–	0.42*
Panamath	81%*	0.36	0.21*	0.15*
Verbal Number Question	62%	0.4	0.32*	0.19*
Point Number (Shape)	87%*	0.85*	0.36*	0.35*
Point Number (Object)	68%*	0.59	0.33*	0.17*
**Spatial Reasoning**	94%*	0.7*	–	0.36*
Verbal Geometry Question	96%*	0.22	0.13*	0.19*
Point Shape	71%*	0.55	0.15*	0.15
Naïve Physics	51%	−0.16	0.01	0.01
Geometric Intruder	61%	0.46	0.19*	0.2*
Map Reading	92%*	0.72*	0.15*	0.17*
**Mental State Reasoning**	72%*	0.14	–	0.12
Point Emotion	76%*	0.31	−0.11	0.15*
Theory of Mind	24%	0.26	−0.11	−0.04
Executive Function	61%	0.73*	–	0.2*
Baseline	74%*	0.77*	0.1	0.09
Response Inhibition	48%	0.82*	0.14*	0.07
Task Switching	30%	0.16	0.16*	0.07
**Overall**	98%*	0.89*	–	0.53*

**Table 5.** Evaluation of the 7.5-year-old assessment battery organized by domain (bolded) and task. Asterisks indicate tasks that meet thresholds for measurement reliability (above chance performance), internal consistency (Cronbach’s alpha), and convergent validity (*p* < 0.003).

**Domain**	**Above Chance (%)**	**Consistency (*α*)**	**Convergent (r)**
**Language**	99%*	0.85*	–
Vocabulary	97%*	0.89*	0.35*
Reading	99%*	0.78*	0.35*
**Numeracy**	97%*	0.76*	–
Panamath	96%*	0.61*	0.32*
Verbal Number Question	69%*	0.6*	0.3*
Point Number	98%*	0.81*	0.38*
**Spatial Reasoning**	98%*	0.64*	–
Verbal Geometry Question	92%*	0.42	0.26*
Point Shape	84%*	0.17	0.24*
Geometric Intruder	99%*	0.59	0.3*
**Mental State Reasoning**	97%*	0.52	–
Emotion Intruder	88%*	0.09	0.15
Theory of Mind	97%*	0.51	0.23*
Gaze Intruder	81%*	0.43	0.2*
**Executive Function**	81%*	0.76*	–
Baseline	78%*	0.79*	0.19*
Response Inhibition	60%	0.79*	0.23*
Task Switching	31%	0.28	0.16
Impulsivity	88%*	0.42	0.2*
**Overall**	98%*	0.91*	–

### 1.5-year-Old Performance

A summary of the evaluation of the 1.5-year-old measures can be found in Table 2.

#### Measurement Reliability.

The vocabulary checklist measures were omitted from this analysis. This is because: 1) the present evaluation focuses on direct cognitive tests of children and this checklist is caregiver reported; and 2) the checklist includes no chance performance measure.

##### Overall.

The Cronbach’s alpha of the entire battery was very low (*α* = 0.16). Less than half of participants performed above chance (46%).

##### Domain.

The Cronbach’s alphas of Executive Function (*α* = 0.13) and Number (*α* = −0.37) were well below the cutoff. Children performed above chance in the Executive Function tasks (78%), but not in the Numeracy task (64%).

##### Task.

The Cronbach’s alpha for every individual task was negative. Only Working Memory met the threshold for above chance performance (79%).

#### Convergent Validity.

##### Task.

For Language, the subscores of the vocabulary checklist were highly correlated (*r* = 0.7). For Executive Function, Attention Switching and Mental Simulation meet the threshold for convergent validity, but not Working Memory.

#### Predictive Validity.

##### Overall.

Overall performance at 1.5 years did not significantly predict performance at 3.5 years (*r* = −0.07).

##### Domain.

Combined performance on Language tasks at 1.5 years predicted performance at 3.5 years (*r* = 0.2), but performance on Executive Function tasks did not (*r* = −0.13).

##### Task.

Productive and receptive vocabulary scores at 1.5 were predictive of performance on the Language task at 3.5 years. No Executive Function task met the threshold for predictive validity, and the average correlation size for every task was negative.

##### Overview of 1.5-Year-Old Measures.

There was little evidence for reliability or validity in the 1.5-year-old tasks, with the exception of the caregiver-reported vocabulary checklist, which met or exceeded thresholds for every evaluative criterion.

### 3.5-Year-Old Performance

A summary of the evaluation of the 3.5-year-old measures can be found in Table 3.

#### Measurement Reliability.

The Block Reproduction task, where children constructed a block tower, was omitted from this analysis, as there was no measure of chance performance that could be defined.

##### Overall.

The battery had a high overall Cronbach’s alpha (*α* = 0.84). Almost all (99%) of participants performed above chance.

##### Domain.

There was a high Cronbach’s alpha for Language (*α* = 0.81) and an acceptable alpha for Number (*α* = 0.66). All other domains demonstrated poor internal consistency. Children performed above threshold in Language (99%), Numeracy (81%), Spatial Reasoning (94%), and Executive Function (97%), but not Mental-State Reasoning.

##### Task.

Only Vocabulary exhibited acceptable internal consistency (*α* = 0.81). All other tasks fell below the threshold for acceptability. Children performed above threshold in 8/11 tasks: Vocabulary, Verbal Number Questions, Point to Number (Symbol), Geometric Intruder, Map Reading, Attention Switching, and Mental Simulation.

#### Convergent Validity.

##### Task.

All tasks in Numeracy and Spatial Reasoning met the threshold for convergent validity, while no task in Mental-State Reasoning or Executive Function did. Due to the fact that only a single Language task was used at this age, no convergent validity metric could be calculated.

#### Predictive Validity.

##### Overall.

Overall performance at 3.5 years was positively associated with overall performance at 5.5 years (*r* = 0.31).

##### Domain.

Performance at 3.5 years was positively associated with performance at 5.5 years for Language (*r* = 0.26), Numeracy (*r* = 0.22), Spatial Reasoning (*r* = 0.21), and Executive Function (*r* = 0.17). No significant association was found for Mental-State Reasoning (*r* = 0.1).

##### Task.

In Language, Vocabulary at 3.5 years predicts later task performance at 5.5 years (*r* = 0.26). In Numeracy, only Point to Number (Objects) met the threshold for predictive validity, with an average correlation of *r* = 0.18. No other individual task met this threshold.

##### Overview of 3.5-Year-Old Measures.

We found that children performed above chance at the overall battery and for most tasks in most domains, outside of Mental-State Reasoning. We also found that overall and domain-level performance was predictive of future performance, again with the exception of Mental-State Reasoning. However, only the Language and Numeracy domains met the criteria for internal consistency. Numeracy and Spatial Reasoning tasks showed adequate convergent validity, but no individual tasks outside of Vocabulary had adequate internal consistency or predictive validity (excepting Point to Number). Thus, we conclude that the 3.5-year-old tasks show some promise as measures of individual differences, but require further development, particularly for Mental-State Reasoning, Executive Function, and Spatial Reasoning.

### 5.5-Year-Old Performance

A summary of the evaluation of the 5.5-year-old measures can be found in Table 4.

#### Measurement Reliability.

##### Overall.

The battery had a high overall Cronbach’s alpha (*α* = 0.89) suggesting internal consistency. Almost all (98%) of participants performed above chance.

##### Domain.

Language (*α* = 0.79), Numeracy (*α* = 0.8), Spatial Reasoning (*α* = 0.7), and Executive Function (*α* = 0.73) all demonstrated adequate internal consistency. In contrast, Mental-State Reasoning had very low internal consistency (*α* = 0.14). Over two-thirds of children performed above chance in Language (100%), Numeracy (88%), Spatial Reasoning (94%), and Mental-State Reasoning (72%), but not Executive Function (61%),

##### Task.

Only 5/15 tasks met the criteria for internal consistency: Vocabulary, Point to Number (Symbol), Map Reading, Baseline Executive Function, and Response Inhibition. More than two-thirds of children performed above chance on 9 of the 15 tasks: Vocabulary, Panamath, Point to Number (Symbol), Point to Number (Object), Verbal Geometry Question, Map Reading, Point to Shape, Point to Emotion, and Baseline Executive Function.

#### Convergent Validity.

##### Task.

All tasks met criteria for convergent validity, with the exception of Naïve Physics, Point to Emotion, Theory of Mind, and Baseline Executive Function. No convergent validity measure could be calculated for Language.

#### Predictive Validity.

##### Overall.

There was a large association between overall performance on the battery at 5.5-years and performance at 7.5-years (*r* = 0.53).

##### Domain.

There were small-to-medium-sized correlations across all domains except for Mental-State Reasoning (*r* = 0.12). Larger correlations were between Numeracy (*r* = 0.42), Language (*r* = 0.42), and Spatial Reasoning (*r* = 0.36); the correlation between Executive Function was smaller but still significant (*r* = 0.2).

##### Task.

For Language, Vocabulary at 3.5 years predicted later performance on Vocabulary at 5.5 years (*r* = 0.36). All Numeracy tasks were predictive of later performance on at least half of related tasks at 5.5 years. For Spatial Reasoning, all tasks except Point to Shape and Naïve Physics met criteria for predictive validity. For Mental-State Reasoning, only Point to Emotion predicted later performance on related tasks. No Executive Function tasks were predictive of later performance.

##### Overview of 5.5-Year-Old Measures.

The 5.5-year-old measures exhibit above-chance performance, internal consistency, and predictive validity overall and in most domains except for Mental-State Reasoning. In addition, Numeracy, Spatial Reasoning, and Executive Function tasks exhibit convergent validity. Especially poor performance was found for Naïve Physics and Theory of Mind, which did not meet the threshold on any evaluative metric. Naïve Physics tested children’s ability to guess which of two objects was heavier based on their movement. Low measurement validity relative to other tasks suggests that children may not have understood the task. Theory of Mind tested children’s ability to suppress their own beliefs and infer another person’s beliefs. Children systematically fail this task at younger ages and succeed at older ages, suggesting that children in our sample may not have reached this point in their social development.

### 7.5-Year-Old Performance

A summary of the evaluation of the 7.5-year-old measures can be found in Table 5.

#### Measurement Reliability.

##### Overall.

The battery had a high overall internal consistency (*α* = 0.91). Almost all participants (98%) performed above chance.

##### Domain.

Each domain showed acceptable internal consistency: Language (*α* = 0.85), Numeracy (*α* = 0.76), Spatial Reasoning (*α* = 0.64), and Executive Function (*α* = 0.76); except for Mental-State Reasoning (*α* = 0.52). Children performed above chance in all domains.

##### Task.

There was acceptable internal consistency on 7/15 tasks: Reading, Vocabulary, Panamath, Verbal Number Questions, Point to Number, Baseline Executive Function, and Response Inhibition. Children performed above chance on all tasks except for Response Inhibition and Task Switching.

#### Convergent Validity.

##### Task.

Across domains, all tasks met the criteria for convergent validity, except for Emotion Intruder and Task Switching.

##### Overview of 7.5-Year-Old Measures.

As a whole, we found high rates of above-chance performance, internal consistency, and convergent validity at all levels of analysis and across all domains in the 7.5-year-old battery.

## DISCUSSION

We find that the assessment battery captures stable individual differences in children’s performance on tasks meant to target their language, numeracy, spatial reasoning, and executive functioning from ages 3.5- to 7.5-years. In contrast, we find evidence of low reliability and validity in tasks targeting children’s mental-state reasoning and in tasks designed for 1.5-year-olds. Moreover, tests targeting cognition in different domains at older ages were not uniformly effective: we find poorly performing individual tasks (e.g., Naïve Physics and Theory of Mind at 5.5 years; Task Switching at 5.5 years and 7.5 years) that should be reworked in further deployments of this battery. Our evaluation contributes to a growing effort to produce formal measures of validity and reliability for novel tools used to evaluate children’s developmental outcomes in field experiments conducted in other cultural settings (Macours et al., 2023).

Despite the limitations of the assessments, the results of our analysis suggest that these assessments represent a promising first step in the use of lab-based experimental methods to study early cognitive development in a markedly different culture. This assessment goes beyond previous developmental assessments, which focus solely on school-related tasks, by attempting to tap into universal cognitive abilities, such as implicit numeracy, navigation, and mental-state reasoning, using experimental methods from developmental cognitive science. We believed these universal skills, which have also been found to predict performance in school-related tasks, would be less sensitive to cultural differences and provide a more flexible set of tools to directly measure children’s cognitive abilities across a range of settings. The effectiveness of these tasks in Ghana (and a subset of tasks in India) open the way for further adaptation in other cultural contexts. We were also encouraged by children’s consistent performance in language and executive functioning tasks, which required more cultural and linguistic adaptation specific to the testing context. However, our particular approach to language adaptation (i.e., beginning with English word lists) was specific to the Ghanaian context, where English proficiency is high and local surveyors were able to provide feedback on the appropriateness of each vocabulary item. Ideally, word lists should either be generated by members of the same communities being tested (e.g., Prado et al., 2018), or with language-neutral methods, such as Swadesh lists (Kachergis et al., 2025). Our approach is not meant to be a substitute for clinical assessment of children’s development, but is instead complementary, providing a strong foundation for tracking individual differences in children following intervention, on skills that predict school-relevant learning later in life. Nor is our battery a substitute for assessments grounded in insights from ethnography or cultural psychology (e.g., Scheidecker et al., 2023), or that include cultural knowledge as a measure in and of itself (e.g., EAP-ECDS from Rao et al., 2019), which can be better adapted to suit the needs of particular communities.

In addition to the usefulness of the battery as a tool for measuring individual differences, the tasks we adapted for use in these communities, particularly those in numeracy and spatial reasoning based directly on experimental paradigms, are a toolkit for studying the emergence of these core cognitive abilities across different cultural settings. Developmental scientists have increasingly looked outside of North American and European populations and into communities where children’s early life experiences are vastly different from those of children growing up in Western countries (Singh et al., 2024). Various early emerging skills including sensitivity to approximate mathematical magnitudes (Lipton & Spelke, 2003), ability to track small numbers of objects in parallel (Feigenson et al., 2002), sensitivity to simple geometric figures and their properties (Dehaene et al., 2006), and navigation by maps (Shusterman et al., 2008) can be tracked in children as young as 3 years of age using portable, language-neutral study materials in noisy, outdoor settings. Finding that these abilities emerge similarly, along similar time frames, suggest that they tap into universal skills. The incorporation of these paradigms in educational interventions also vastly increases the scale that research in cognitive development can be conducted. For instance, while Pica et al. (2004) were able to track the development of numerical reasoning in 29 children from an Amazonian village, the current study examined the same skills in over 1200 children.

There may also be important lessons to be learned in the tasks that performed poorly. For example, children’s performance on Theory of Mind was low at 5.5 years but much higher at 7.5 years. This finding might reflect problems with its implementation at 5.5 years. However, previous reviews have also found that the age at which children achieve above chance performance on false-belief tasks differs greatly across countries (Liu et al., 2008; Wellman et al., 2001). There are also cultural differences in how early children are capable of making certain inferences about the mental states of others. For example, children from more individualistic societies perform better on tasks requiring them to understand that other people can have different beliefs about the same object than children from more collectivist societies. In contrast, children from more collectivist societies perform better on tasks requiring them to understand that other people can be either knowledgeable or ignorant about an object (Wellman, 2014). It is possible that our results point to cultural differences in the timing and trajectory of children’s emerging theory of mind in Ghana, which could be examined in future studies.

### Addressing the Challenges of Infant Assessment

Three potential advantages of using tasks developed in labs devoted to early cognitive development as a means of testing infant cognition in the field, highlighted in the Introduction, are the presence of these abilities early in infancy, their persistence into adulthood, and their role in guiding children’s learning. We find, however, that most of the cognitive tasks for 1.5-year-olds perform poorly compared to the tasks presented to the other age groups. Because survey administration was not videotaped and reviewed, we cannot determine whether surveyors encountered more difficulty with children in this age group. However, there are two inherent problems with behavioral testing of infants in any setting. First, younger children respond more slowly on cognitive tasks than do older children, and they more quickly become bored if a task goes on for too long. As a result, few trials can be given to infants, whereas progressively more trials can be given to older children. Second, younger children respond less reliably than older children with increases in the number of response options they must consider. As a result, the present battery presented infants with only 2 or 3 response alternatives, with the number of alternatives increasing at each successive age.

Behavioral testing of infants in low- and middle-income countries is even more difficult because of how infants’ responses may be shaped by proximal factors. Previous research has found that parents with and without school exposure have distinctive patterns of interaction with their children beginning at birth (LeVine et al., 2012). Cultural norms may also influence how children behave during testing. For instance, Number Comparison tests children’s ability to distinguish between larger and smaller amounts by presenting them with different piles of toys and asking, “Which would you rather play with?” In typically laboratory settings, it is assumed that children will prefer to play with more toys rather than fewer. But this assumption might not hold if children are raised in cultures where it would be considered more polite to take fewer toys (and thereby leave more toys for others). Similarly, the Attention Switching task tests children’s ability to find a hidden toy under a cup after having their attention diverted to a distractor cup by the surveyor pointing. But for children raised in cultures where they are expected to be deferential to adults, they may interpret the point to the distractor cup as an instruction to look at it, which may create confusion when they are given a conflicting instruction to not pick that cup. Because these children are too young to understand complex instructions or elaborate on their actions, it is difficult to determine whether poor performance reflects their limited executive functioning or a misunderstanding of the pragmatics of the task.

The early emergence of cultural and socioeconomic influences on parent behavior also calls into question whether tasks based on infant experimental paradigms actually measure more universal forms of development. The research our tasks are based on, has found that 1) representations of objects, actions, places, and people emerge at birth, and 2) the systems that support them remain present and functional throughout life, as attested by research on diverse populations of adults with minimal formal education (Dehaene et al., 2006; Pica et al., 2004). This does not imply, however, that early experience has no effect on children’s learning and development. In fact, the field experiment that generated the data used in this paper found that offering high school scholarships to female students resulted in their children scoring higher on our assessment battery. Nor is it incompatible with the possibility that tasks may differ in their efficacy across cultures or geographies. As an example, we would expect greater task validity and reliability in communities where infant can be tested indoors, regardless of culture, due to their insulation from noise, inquisitive neighbors, and other outdoor distractions. Our motivation for using these paradigms as a basis for cognitive assessment is that the abilities they tap into are *present* in all children in the first place, such that we can use them to measure the effects of the early environment on development in vastly different settings.

A possible solution to many of these problems is to use tasks that rely on automatic responses. For example, infants and toddlers can be tested on many more trials on tasks that use looking time to gauge their ability to track objects or detect changes in visual displays (e.g., Dillon et al., 2020). Richer data can also be obtained from infants by using neural measures of brain activity in response to numerical, spatial, or social events (e.g., Fló et al., 2022). These methods remove all action demands, sharply reduce the duration of each trial, and can be made language- and culture-neutral. Some methods, like EEG, can also deliver stimuli to sleeping infants, revealing sensitivity to language (Fló et al., 2022) and number (Gennari et al., 2023). The speed of data collection using these methods could also make it possible to test young children across more domains, offering a fuller picture of cognitive development. Some of these approaches have already been tested in LMICs with success (Lloyd-FoDuflo et al., 2019). By measuring brain activity during passive viewing or listening, future cognitive batteries may overcome many of the limitations described above.

### Culture and Context in the Development of Early Childhood Assessments

Many of the tasks used in this battery were chosen because laboratory-based studies of infants and studies of adults in diverse cultures provide evidence that the abilities they tap are universal and are therefore applicable to children living in any culture. Other tasks, such as receptive vocabulary, were used due to their successful adaptation in other African countries (e.g., Pakendorf & Alant, 1997 in South Africa; Zhu et al., 2025 in Kenya). However, a number of cultural and contextual factors may have influenced children’s performance on different tasks and compromised the tests’ validity, despite our best efforts to adapt them for use in this context. These factors are also potential obstacles to using the battery in other LMICs, where children may be raised in vastly different environment that the one studied here. While we do not give an exhaustive list of all potential cultural confounds (see Jukes et al., 2024 for a more in-depth review), we believe the following factors are especially pertinent for both interpretation within our sample as well as adaptation to other LMICs.

First, previous tests of children in African countries have found that children are less comfortable engaging with unfamiliar adults than are children in Western countries, even those from the same regions (Otto et al., 2014). While we attempted to address these issues by enlisting the child’s caregivers to help in some tasks, engagement with an adult outside of the community is inherent to all assessments in this battery. In communities where children do not typically engage proactively with any adult, let alone unfamiliar ones, these methods run the risk of underestimating children’s knowledge. This might have particularly impacted 1.5-year-old children, who would have the least experience engaging with adults outside of their family and community. We might also expect this to be a more significant influence on performance for children who live in relatively more remote regions situated farther away from urban centers. Similarly, children may be less able to interpret social stimuli, such as emotions or eye gaze, using pictures of people outside of their ethnic or cultural groups. This is one possible reason for consistent under-performance on many of the mental-state reasoning tasks, which presented faces of people whom the children did not know. Thus, future deployment of the battery may need to enlist the help of surveyors to create social stimuli that children are familiar and comfortable with.

Second, children’s performance on these tasks might also be influenced by their familiarity with different forms of media. Zhu et al. (2025) found that in urban Kenya, children’s performance on a picture-based word learning task was related to their exposure to pictures in books. Many families in our sample do not have books in the home (565 out of 1198 reporting) and consequently children may have been less comfortable engaging with printed materials. Children’s familiarity with technology also seemed to differ not only across homes but also with age. During piloting, we found that 1.5-year-olds and 3.5-year-olds were uncomfortable engaging with laptops. This discomfort was attributed to their unfamiliarity with these testing mediums. As a result, tasks for 1.5-year-olds were conducted mainly with physical objects (e.g., animal figures, small building blocks) and tasks for 3.5-year-olds were given more tasks with printed cards. While 5.5- and 7.5-year-olds showed more comfort with the laptops, and thus were administered tasks electronically, some children may have been relatively less familiar with them, which could have impacted their performance. The lack of uniformity of task delivery across ages may also have contributed to larger longitudinal correlations between performance on tasks from 5.5- to 7.5-years-old compared to 1.5- to 3.5-years (objects to cards) and 3.5- to 5.5-years (cards to laptops). Children’s familiarity with different forms of media and with different technologies is likely to vary significantly both within and outside of Ghana; thus, researchers should develop modes of task delivery that are appropriate for their local contexts.

Finally, children’s variable experience in preschool environments may influence the effectiveness of the battery. Formal schooling has been found to exert a tremendous influence not only on the pace of children’s development, but also on how children approach learning and problem-solving (e.g., Greenfield & Bruner, 1966; Luria, 1976; Scribner & Cole, 1973; for broader review, see Kroupin et al., 2024). Children who attended preschools or primary schools would have been accustomed to performing tasks and answering questions, posed by an adult outside their immediate family, of the sort used in the present cognitive batteries. In contrast, children with no school experience may have been puzzled or perturbed by the approach of a stranger who questioned them. Because the youngest children were most likely to lack any school experience, their performance on the cognitive battery may have been affected by this unmeasured source of variability. The lower success rates in younger children, found in prior studies using direct cognitive testing of children (Fernald et al., 2017), may have occurred, in part, for this reason. Differences in education may also influence the generalizability of the battery to other countries. Over the years devoted to cognitive testing, Ghana held the highest enrollment rates in early childhood education programs in Sub-Saharan Africa (UNICEF, 2019). As a result, children in our sample may have been particularly receptive to our tasks than would otherwise be expected in another LMIC where preschool attendance is lower.

### Establishing Other Forms of Assessment Validity

As we previously noted, the length of the assessments and general survey precluded our testing of children on other assessments, such as the IDELA. This limits our ability to establish concurrent validity, except by checking for correlations between scores on related tasks within age groups. Similarly, we lack standardized academic assessments, which could be used to establish the relationship between task performance and success in school. Prior studies have found that performance on the types of tasks included in our battery predict performance on related tasks in school (Halberda et al., 2008). However, we could not find any studies linking these factors in populations living in LMICs. Establishing this relationship is especially vital for the use of these tasks to evaluate RCTs in early childhood education.

The present analysis also cannot distinguish between domain-specific and domain-general factors influencing performance on this battery. Because cognitive development across different domains is driven by uniform maturational processes, we expect performance to be somewhat interrelated regardless of domain. While the assessments show robust within-domain intercorrelations, this kind of analysis ignores how correlated tasks within one domain are to tasks within another domain. For example, should correlations between the language measures be taken as evidence that we are tapping into language ability if they are more related to performance on the Number measures than to each other? Such measures of divergent validity are absent from the present paper.

A difficulty in estimating this metric is that there are many reasons to expect there should be intercorrelations between tasks in different domains, outside of a child’s general interest or motivation to complete the battery. For example, within language research, meta-analyses have revealed consistent correlations with social cognition (Milligan et al., 2007), executive function (Follmer, 2018), and mathematics and geometry (Peng et al., 2020). In the battery, implicit numeracy tasks such as Panamath have been related to children’s later performance on tasks using numerical language (Feigenson et al., 2013; Gilmore et al., 2007). The degree of interrelatedness is also likely to be different from task to task. For example, it is possible that the Point to Shape task, testing 5.5-year-old children’s ability to match a shape name with the appropriate visual form, is more related to later performance on the Reading task than to the Point to Shape task that presents the same children with solid objects. In order to distinguish domain-specific associations between the tests in this battery from domain-general associations, we would need to know how large to expect within- and between-domain correlations between tasks to be. These correlations might be estimable from previous validations of similar assessments, as well as from the meta-analytic studies listed above. For the purpose of the present analysis, we have focused on establishing validity within each cognitive domain, both concurrently and over time.

## CONCLUSIONS

Research in developmental cognitive science has produced a rich array of lab-based paradigms for studying cognitive abilities present in childhood across cultures. In this paper, we present an open-source assessment battery based on this research for use in low- and middle-income countries. Data collected from in Ghana suggests that the assessment battery is sensitive to individual differences in performance in children as young as 3.5 years, and it is increasingly effective at later ages. The battery exhibits adequate measurement reliability, convergent validity, and predictive validity overall and on most domains and measures. We conclude that targeting areas of cognitive development that have been found in laboratory studies to emerge and grow in early childhood, and have been shown in field studies to be present in adults in diverse cultures, can capture meaningful variance in children’s performance, and therefore can be used to evaluate field experiments in child development and education. These assessments also offer a viable means of conducting field research in developmental cognitive science in non-Western populations at a much larger scale than typically possible. Nevertheless, the sensitivity and specificity of direct cognitive tests tend to be higher for older children, who have more exposure to adults outside their immediate families, and who can be tested on more trials with more alternative responses. There are also outstanding questions about the appropriateness of these tasks for adaptation to other settings, which may need to address differences in cultural attitudes towards adult-child interactions, familiarity with testing mediums, and differences in exposure to formal schooling. Future adaptations should also attempt to establish convergent validity with other testing batteries and with standardized school assessments, as well as divergent validity between tasks in different domains. We have made all data and materials available publicly to encourage further iteration on these measures and adaptation to other LMICs.

## ACKNOWLEDGMENTS

We would like to thank the members of the Spelke Lab who contributed to the development of the testing measures: Adrien Maries and Brandon Woo. We would like to thank our collaborators at the Abdul Latif Jamil Poverty Action Lab: Esther Duflo, Pascaline Dupas, Mark Walsh, Erin Grela, Gabriella Fleischman, and Flora Bergounhon. We would also like to thank the staff of Innovations for Poverty Action in Ghana for providing exemplary training and research assistance: Stephanie Adjovu (Research Coordinator), Renaud Comba (Policy Manager), Kwame Akakpo (Research Associate), and Isaac Arthur (Senior Research Quality Associate). We would like to thank all our surveyors for their years-long work on data collection: Belinda Bakah, Vivian Ahiatornyo, Mercy Chidi Atanley, Augustina Quarshie, Mabel Akorfa Quame-Amaglo, Gifty Amaney, Anette Fafa Amegayi-Senaya, Stellina Pana, Reindorf Appiah, Kawamu-Din Ibrahim, Jonathan Tetteh, Amankwah Maxwell.

## FUNDING INFORMATION

The study protocol was approved by the Ghana Health Service Ethics Review Committee, and the IRBs of MIT, Stanford, and IPA. The funding for this study was provided by the British Academy, the JPAL Post-Primary Education Initiative, and USAID-DIV. This study falls under the Harvard IRB-approved protocol “Ghana Children and Caregiver Panel Survey” (SITE 17-0388), which itself is an extension of the MIT IRB-approved protocol “Measuring Returns to Secondary Education” (#0610001931). This study was preregistered in The American Economic Association’s registry for randomized controlled trials under RCT ID AEARCTR-0000015.

## AUTHOR CONTRIBUTIONS

J. C.: Conceptualization, Formal analysis, Methodology, Validation, Visualization, Writing – original draft. E. S.: Conceptualization, Supervision, Writing – review & editing.

## DATA AVAILABILITY STATEMENT

All testing materials and survey data are accessible through Open ICPSR: https://doi.org/10.3886/E241205V1.

Supplementary materials, including additional analyses and code, can be accessed online through the Open Science Foundation: https://osf.io/fxzcu.

## Note

^1^ Beginning in July 2021, 346 children between the ages of 30 and 36 months were administered a new set of tasks. The validity of these tasks will be addressed in a subsequent publication.
